# Somali and Eritrean parents experiences and challenges in the diagnosis and early intervention of autism spectrum disorder in Norway

**DOI:** 10.1186/s12887-026-06765-y

**Published:** 2026-03-26

**Authors:** Abdi Gele, Hodan A. Duale

**Affiliations:** 1 Department of Health Service Research, Norwegian Institute of Public Health, Post-box 222, Skøyen, Oslo 0213 Norway; 2 Department of maternal and child health, Somali Institute for Health Research (SIHR), Hargeisa, Somaliland

**Keywords:** Autism, African immigrants, Norway, Early diagnosis, Early interventions, Eritrean, Somali

## Abstract

**Background:**

Children of immigrants in Norway have a higher risk of being diagnosed with autism spectrum disorder (ASD) compared to non-immigrant children. However, some immigrant groups may receive a late diagnosis, thus missing out on beneficial early interventions. The aim of this study is to explore parents’ experiences with the early intervention for autism in Norway.

**Methods:**

We conducted 15 in-depth interviews (IDIs) with immigrant parents of children with autism living in five different cities across Norway. The interviews were held between November 2023 and February 2024. The study information was shared through social media (Facebook) in Somali and Tigrinya. Nine participants contacted the researcher to confirm their willingness to participate in the study, while the remaining six participants came through snowballing. The interviews were conducted in Somali for Somalis or Tigrinya for Eritrean participants. The interviews were audio recorded and transcribed verbatim by the interviewer and research assistant. Thematic analyses were used for the data analysis. The data analysis was guided by the Tanahashi framework of health service coverage.

**Results:**

Four key themes emerged from the interviews, which were organized according to the Tanahashi framework: Parents’ perception of the availability of early intervention services; how well the interventions aligned with family expectations; the quality of the partnership between parents and service providers; and the challenges related to late diagnosis. Overall, the participants were predominantly satisfied with the interventions provided for their children. However, they expressed significant dissatisfaction with diagnostic services, indicating that this aspect was a major source of discontent.

**Conclusions:**

Long waiting times for autism diagnosis can cause considerable stress and anxiety for parents, as they delay the child’s access to necessary interventions. It is crucial that healthcare providers listen to and address parents’ concerns about their child’s development effectively and promptly. Further research is needed to understand the level of satisfaction with the intervention among parents with lower levels of education and those who are not proficient in the language of the host country.

**Supplementary Information:**

The online version contains supplementary material available at 10.1186/s12887-026-06765-y.

## Background

Autism spectrum disorder (ASD) includes childhood autism, atypical autism, Asperger syndrome and pervasive developmental disorders. This continuum of disorders ranges from severe to mild, and they are heterogeneous both in etiology and clinical phenotype. Autism impacts social communication, interaction, interests and repetitive behaviours that can sometimes be detected at 18 months of age or younger by professionals [1], while parents can begin to recognize characteristics of ASD as young as the first year of the child’s life [2].

There is increasing evidence that immigrant children are at greater risk of developing neurodevelopmental disorders, particularly ASD [3–8]. Research in different Western countries shows a higher prevalence of ASD, specifically, for children of East African parents [3, 9, 10]. For example, an estimated 0.10% of children in Norway are diagnosed with ASD [11], while 0.74% of immigrant children are diagnosed with autism [12]. A soon-to-be-published study showed that while children with immigrant parents had a greater risk of being diagnosed with autism, children with Eritrean and Somali parents were among those with the highest risk of being diagnosed with autism in Norway [13].

Evidence indicates that immigrant children experience delays in diagnosis and face increased difficulties accessing appropriate healthcare [14, 15]. These delays are particularly concerning because autistic children can miss out on early interventions, which are highly beneficial for their clinical prognosis [16]. Prompt diagnosis and early entry into intensive, comprehensive, and appropriate intervention services for children with ASD have been widely endorsed [17]. Early interventions can enhance autistic children’s communication, learning, and social interaction skills, taking advantage of the neuroplasticity of their brains at a young age [18]. The care and support for children with ASD require a multifaceted approach, including diagnosing disorders, assessing children’s capacities, designing clinical and educational interventions, and continually addressing the evolving needs of the children [19].

The therapeutic services available to children with ASD address speech and communication, social skills, self-care, independence etc., and interventions typically start as early as two years of age. On the other hand, parental involvement in the intervention covers a broad range of activities, from parent training and homework routines to participation in intervention design and implementation [20]. Research suggests that parental involvement in treatment improves the generalizability of skills and increases the amount of intervention the child receives [21]. Siller et al. stated that parental involvement is relevant because parents must replicate strategies within the home [22]. However, families and professionals often report difficulties in collaboration that are frequently related to opinion divergence and communication barriers, resulting in the struggle of support teams to coordinate individual efforts to best support children with ASD [19]. Parents are regarded as having the most powerful influence during early childhood; thus, their inclusion and active participation in interventions are crucial [23].

A study that explored immigrant and refugee mothers’ perceptions of barriers and facilitators for mental health care for their children revealed that lack of information, language barriers and feeling unheard by service providers serve as barriers for mental health care for their children [24]. Navigating through layers of care involved in helping a child with ASD demands a greater capacity for adaptation for families who have left their social capital and their country of origin behind [25]. Given the central role that parents play in early ASD intervention, gaining an understanding of parents’ experience and their perceptions of intervention success is a critical research goal [26]. Nonetheless, the experiences of immigrant parents of children with autism in Norway and their satisfaction with the early interventions provided to their autistic children have not been studied before. The aim of this study is to explore parents’ experiences with the early intervention for autism in Norway.

## Methods

### Study participants

Fifteen in-depth interviews (IDIs) with immigrant parents of children with autism were conducted in five different cities across Norway. The interviews were held between November 2023 and February 2024. Except for one second-generation immigrant mother with Somalia-born parents, all participants were born either in Somalia or Eritrea. We included parents whose children had been diagnosed with autism by a doctor, and we excluded parents of children with other developmental disorders.

### Recruitment and data collection

Fifteen parents (Table 1) were recruited for the study by the first author and assistant. The study information was shared on social media platforms such as Facebook, in Somali, Norwegian, and Tigrinya languages. Nine participants directly contacted the researcher to confirm their willingness to participate in the study, while the remaining six participants were recruited through the snowball sampling method.

We used a question guide with semi-structured questions for the interviews. These questions were designed to elicit detailed responses from the participants regarding their experiences and satisfaction with early intervention and other services provided to children with autism.

Before starting the data collection, each participant received information about the study in their preferred language and at a location convenient for them. The interviews were conducted in Somali for Somali participants and in Tigrinya for Eritrean participants. Twelve interviews were transcribed in Somali, while three interviews that were taken in Tigrinya was transcribed in Norwegian. The reason was that the Eritrean research assistant could speak fluent Norwegian, and very good English, but it was quicker for her to write the transcripts in Norwegian than English. Finally, all the transcripts were translated into English through forward translation by the multilingual authors. We preferred forward translation given the competence of authors in the local culture of immigrants and the language used for the interviews [27].

**Table 1:** Characteristics of study participants and their children

Parents’ characteristics				Their children’s characteristics				
*N*	Relation	Education	Born	*N*-Children	Born	Diagnosed with ASD	Age of the child with ASD	Sex
1	Mother	secondary	Somalia	6	Norway	2	8 &14	Boys
2	Mother	University	Norway	2	Norway	1	5	Boy
3	Mother	University	Somalia	2	Norway	1	8	Boy
4	Mother	University	Somalia	3	Norway	1	4	Boy
5	Mother	University	Somalia	3	Norway	1	20	Boy
6	Mother	University	Somalia	5	Norway	1	14	Boy
7	Mother	Secondary	Somalia	3	Norway	1	6	Boy
8	Mother	Secondary	Somalia	4	Norway	1	6	Boy
9	Mother	No education	Somalia	2	Norway	1	17	Boy
10	Mother	Secondary	Somalia	4	Norway	1	17	Boy
11	Father	University	Eritrea	3	Norway	1	9	Girl
12	Father	Primary	Eritrea	8	Only 1 born in Norway	1	6	Girl
13	Father	Primary	Eritrea	2	1 born in Norway	1	6	Boy
14	mother	University	Somalia	2	Norway	1	7	Boy
15	mother	primary	Somalia	2	Norway	1	24	Boy

### Framework for data analysis

The Tanahashi framework evaluates four key dimensions of service delivery: availability, accessibility, accommodability, and acceptability [28]. This framework was used to identify service gaps and explore strategies for achieving more equitable coverage of autism services among African immigrant communities in Norway. Our analysis focused on how these four dimensions specifically impact the access to and utilization of autism services by immigrant communities. Availability, defined as the provision of sufficient services, was assessed by evaluating how well the services meet the needs of the African immigrant community in Norway. Accessibility, involving the absence of barriers, was examined by exploring how easily African immigrants can access these services, with particular emphasis on prompt diagnosis. Accommodability, meaning the tailoring of services to user needs, assessed the alignment of services with the community’s needs, constraints, and preferences, including addressing language barriers. Acceptability, focusing on cultural respect, was determined by evaluating how comfortable the community feels with the services’ characteristics and their ability to adequately address cultural sensitivity and expectations. The aim was to comprehensively address the experience of African immigrants with autism interventions, paving the way for more effective and inclusive healthcare strategies.

### Data analysis

**Fig. 1:**
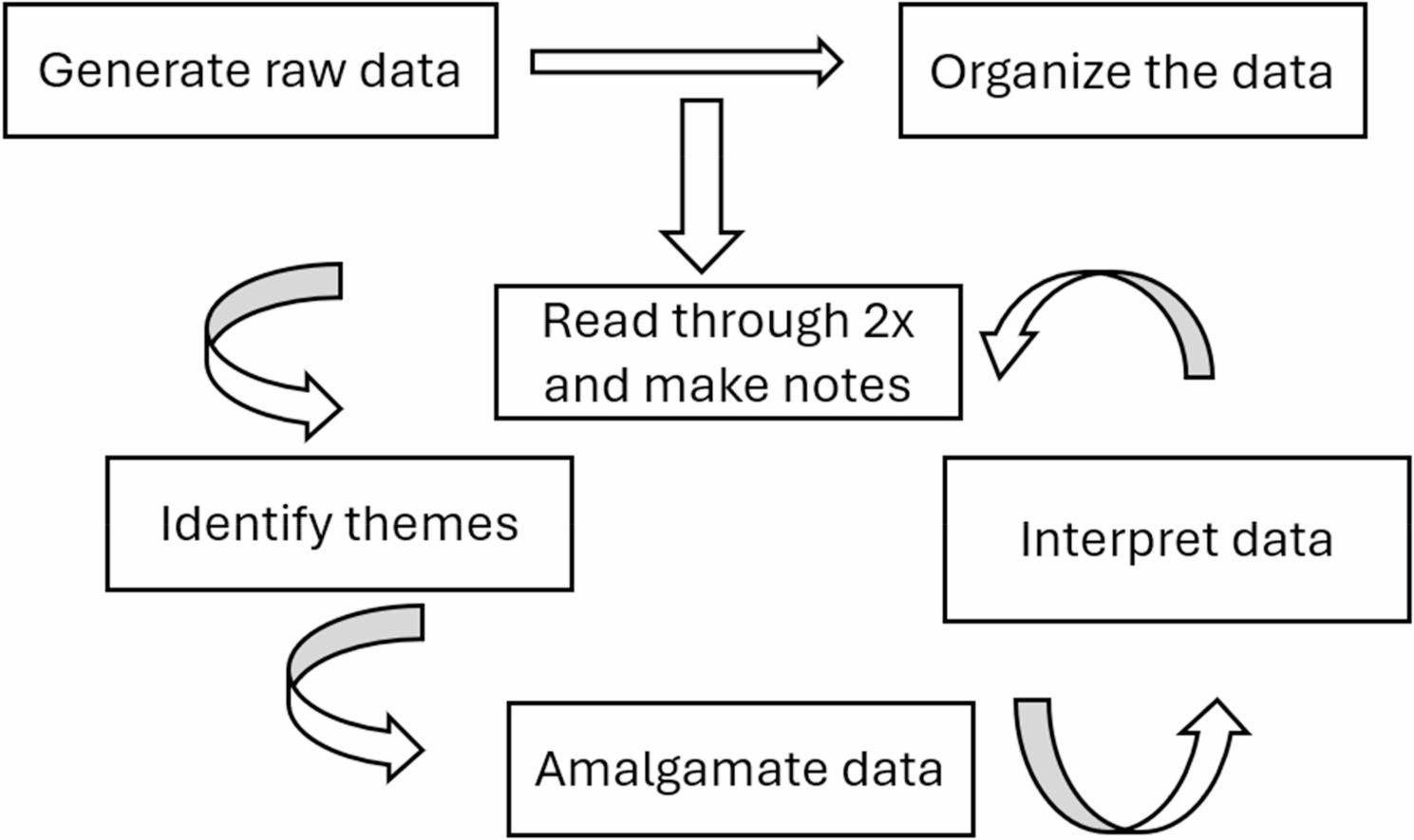
Steps in thematic analysis

The data were analyzed using thematic analysis, following the guidelines established by Braun and Clarke [29]. Initially, the interview transcripts were closely reviewed to foster a deep understanding of the data and to immerse in the parents’ narratives (Fig. 1). Initial codes were then generated to identify key patterns and recurring topics related to their experiences. These codes facilitated organizing the data into meaningful segments. Subsequently, a meticulous examination of the codes led to the identification of overarching themes that captured the essence of the parents lived experiences regarding interventions provided to their children. Once the themes were clearly defined, a comprehensive review was conducted to ensure they accurately reflected the data, with adjustments made as necessary. Finally, the themes were refined and named, providing detailed descriptions that effectively conveyed the insights gathered from the parents’ stories. Throughout this process, a systematic approach was maintained, ensuring a thorough and insightful analysis. NVivo software (version 14) was utilized for the data analysis.

A wide range of experiences and perspectives from the diverse in-depth interviews were verified against each other, to help ensure the trustworthiness of the study results. Following the recommendation of employing a minimum of two methods of rigour within qualitative research [30], we conducted member checking, which is a practice to increase study credibility and confirmability that involves asking a research subject to verify the transcription of an interview [31]. Participants were asked to verify the completeness and accuracy of the transcript to ensure that the transcript truthfully reflected the meaning and intent of the participants’ viewpoints. We also conducted investigator triangulation and reflexivity to ensure the scientific rigour of the current study [32]. Two investigators were involved in the process of data analysis, which allowed different perspectives and ideas. We received ethical approval from the Norwegian Institute of Public Health (eProtokollnr: 4192–4192). Full information about the study was given to participants prior to data collection. Participants were informed that they could withdraw from the study at any time. Participants’ attitudes toward ethical conduct are vital to a relationship likely to produce high-quality data [33]. During the research process, confidentiality of the participants were ensured by presenting the results in a manner that prevents any identification of individual participants.

## Results

The study themes and sub-themes are summarized in Table 2. Four themes and seven sub-themes emerged from the interviews. In general, except for one parent, the participants were predominantly satisfied with the interventions provided to their children. Diagnostic services were the only service for which participants were largely discontented.

**Table 2:** Themes and subthemes categorized within the Tanahashi Framework

Tanahashi framework	Availability	Acceptability	Accommodability	Accessibility
Main themes	1- Early Interventions Through Parents’ Lens	2- Alignment of Interventions with Family Expectation	3- Parent–Professional Partnership	4.Delayed diagnosis
Subthemes	A Range of Intervention Strategies	Contentment with Provided Interventions	Providers Exhibited a Collaborative Spirit	Neglect of Early Parental Concerns
		Mixed reactions	Language and knowledge as key to collaboration	Parents’ proposed improvements

### Theme 1. Early interventions through parents’ lens (Availability)

#### A range of intervention strategies

The experiences shared by Participants highlighted the multifaceted support systems in place for children requiring specialized educational and therapeutic interventions. Incorporating the framework of healthcare availability into the discussion of the participant experiences highlights the complexity and multifaceted nature of support systems needed for children requiring specialized interventions. Participants illustrated comprehensive support frameworks involving educators, therapists, and pedagogues coming together to address the education, social, and emotional needs of children. The availability of such multifaceted support systems plays a critical role in determining the comprehensive care a child receives. The engagement of educators, assistants, and specialists in structured settings, like schools and therapeutic programs, is fundamental in leveraging developmental resources. The inclusion of extracurricular activities, such as swimming and music therapy, broadens the scope of available interventions, recognizing the essential role of diverse therapeutic avenues in nurturing a child’s holistic growth beyond traditional services. While participants appreciated the available support, they also indicated concerns regarding the quality and effectiveness of service delivery, highlighting a key aspect of healthcare availability: it is not sufficient for services to be merely present; they must also be of high quality.

My son received behavioral and language therapy, along with 25 h per week of Pedagogic Psychological Service (PPT). . We receive substantial assistance; without it, my son wouldn’t be where he is today. However, the quality of this help is another matter. (Participant 14)

*My child was supported by a special pedagogue and two additional helpers. He also attends swimming and music therapy sessions. (Participant 2)*

Some parents also reported receiving additional types of support in special cases where it was critically needed. This included the assistance of a personal aide who helped the child during weekends. This support provided much-needed respite for the family and ensured continuous care and development for the child outside of regular service hours. The presence of a personal aide allowed parents to take breaks and manage other responsibilities, while also giving the child consistent support and opportunities for growth.

*My son travels to and from school by taxi. Additionally*,* an assistant takes him to outdoor activities on weekends and occasionally to children’s clubs. (Participant 9)*

A participant highlighted the proactive steps taken by parents to better support children with autism, emphasizing the importance of education and training in effective caregiving strategies. Attending special courses equips parents with essential knowledge and skills needed to understand their child’s unique needs and foster an environment conducive to growth. This initiative reflects a commitment to not only enhancing the child’s well-being but also empowering the parent with the confidence and ability to effectively navigate challenges associated with autism. The participant’s involvement in such educational opportunities underscores the role of tailored training programs in bridging knowledge gaps, potentially reducing stress and improving caregiving outcomes.

*I attended special courses that trained me on how to care for a child with autism (Participant 13)*.

### Theme 2. Alignment of interventions with family expectation (acceptability)

#### Contentment with provided interventions

Parents were asked about their satisfaction with the interventions provided to their children with autism. Participants’ narratives highlighted the importance of support systems that are holistic and family-centric. They emphasize that interventions should cater not only to the child’s developmental needs but also consider the overall family dynamic, enhancing both child outcomes and parental opportunities. The enhanced confidence, independence, and opportunities described by participants advocate for an approach that views the family as an interconnected unit, where positive changes in one area can significantly influence and benefit the whole. Framing healthcare acceptability within this family-centric narrative suggests a shift towards viewing parents and families as full partners in care. When systems align with this perspective, they are more likely to facilitate successful, sustainable interventions that respect and leverage the critical role of the family in achieving optimal outcomes for children with autism.

*My child attended nursery and then school*,* and we received substantial support. I believe the help he received was impressive; without it*,* I wouldn’t have been able to pursue my own education. They educated and assisted me*,* and I deeply appreciate that. (Participant 5)*

Almost all participants expressed that the interventions significantly improved their children’s situation. Parents reported various ways in which their children had developed and progressed. One common theme among the responses was a noticeable increase in the child’s confidence. Additionally, parents observed improvements in communication skills, better interaction with peers and adults, enhanced learning abilities, and greater personal independence. Many parents shared specific examples, such as their children being more comfortable in social settings, more adept at expressing their needs, and becoming more self-reliant in daily activities. However, one respondent noted that parents are satisfied with the services they receive, regardless of the quality, because their point of reference is their home country, where such services are not available. They appreciate any support provided, driven by the belief that these interventions would not have been possible for their children if they were in Somalia or Eritrea.

*Our satisfaction is based on our experiences from our home country*,* Somalia*,* where such services are non-existent. Although the services we receive here are better in comparison*,* they are not of the highest quality. I am not happy with the quality. Sometimes*,* the person who has established a relationship with the child might leave the job or take long-term leave. Children with autism thrive on routines*,* and interruptions can reverse their progress and make it difficult for them to adapt to changes. I believe the municipality should implement preventive measures to ensure the continuity of routines is maintained. (Participant 14)*

#### Mixed reactions

Other parents expressed mixed reactions regarding whether the interventions fully met their needs and expectations. While participants were generally pleased with the support and services provided, they also encountered some shortcomings that fell short of their expectations. Some parents appreciated the overall positive impact on their children’s development, including improvements in social skills, communication, and daily functioning. However, they also pointed out areas where the services could be enhanced. For instance, parents noted issues such as a need for better training and sensitivity among staff members to ensure high-quality care. These mixed reactions highlight the importance of addressing both the successes and the areas in need of improvement to ensure that the interventions more comprehensively support the needs of children with autism and their families.

*The intervention did not fully meet my expectations. I am 80% satisfied with the support*,* but 20% of my expectations remain unmet. As a mother*,* I want the best for my child. The experts who help my child tend to focus only on his differences from other children*,* viewing him primarily through the lens of his autism diagnosis. My expectation was that they would see and treat him as a whole person*,* not just as someone with autism. While I appreciate the help*,* it would have been better if interacted with my child as a child*,* rather than solely as an autistic individual. (Participant 14)*

### Theme 3. Parent–professional partnership (accommodability)

#### Providers exhibited a collaborative spirit

The concept of accommodability in both educational and healthcare contexts emerged as a central theme from participant narratives. Participants highlighted the importance of collaborative approaches within the Pedagogic Psychological Service (PPT) and related interventions that actively involve parents in every step, emphasizing a partnership model that values parental insights and fosters collaborative decision-making. Such practices not only empowered parents but also enhanced the effectiveness of intervention plans by tailoring them to the child’s specific needs. This inclusive strategy built trust and cooperative relationships between parents and professionals, leading to more successful developmental and educational outcomes. The active engagement and respect for parental input contributed to satisfaction and ensured that interventions are responsive and accommodable to the child’s unique circumstances.

*The professionals who provided the Pedagogic Psychological Service (PPT) kept me fully informed and involved in every step of the process. They would send me drafts of their reports and ask for my input*,* allowing me to suggest changes or additions. We worked together very effectively. Both the nursery school and the PPT staff were extremely collaborative. (Participant 7)*

*I was included in every aspect of my child’s treatment. Whenever there was a meeting about my child’s situation*,* I was invited to participate. My suggestions were always taken into consideration*,* and this consistent involvement has been in place since he was diagnosed. I am happy with the way I have been included in my child’s treatment. (Participant 15)*

One of the parents emphasized the importance of parents being proactive in engaging with service providers and initiating collaboration. According to this particular participant, parents possess an intimate understanding of their child’s needs and behaviors. As such, they should not merely wait to be included in the decision-making process but should actively seek and demand collaboration to ensure that their child receives the best possible care. This proactive approach allows parents to share their unique insights and advocate for interventions that are tailored to their child’s specific needs. By taking the initiative to engage with providers, parents can foster a more effective and responsive partnership, which ultimately benefits the child’s development and well-being. This parent strongly believes that their active involvement is crucial in shaping a supportive and comprehensive intervention strategy.

*I am often involved in designing my child’s daily routine*,* but I believe it is crucial for parents to actively seek collaboration and provide information about their child’s needs to professionals. If parents do not take the initiative*,* the people helping the child may not step up either. When parents involve themselves*,* they are more likely to be included in the process. (Participant* 3)

### Language and knowledge as key to collaboration

One of the participants stressed the complexities surrounding parental involvement in a child’s care within educational and psychological support systems. While the participant expressed satisfaction with their involvement and the responsiveness of professionals to their suggestions, the narrative revealed systemic barriers that can impede access for some parents. The participant acknowledged their ability to actively demand involvement, crediting their education, language proficiency, and understanding of the system as key factors that facilitate this engagement. This implies that without these skills, parents might struggle to assert their role in their child’s care, reducing their influence over the process. It highlighted an existing inequity where those who lack language skills or familiarity with the system might be excluded from meaningful participation, suggesting a need for systemic changes to ensure inclusivity. The participant underscored the importance of efforts to bridge language and education gaps, thereby empowering all parents to advocate for their children’s needs. Participant’s experience draws attention to the critical need for an environment where all parents, irrespective of language or educational background, can equally contribute to their child’s care.

*I was involved in my child’s care*,* and I also actively demanded it. Most of the time*,* my suggestions are respected and considered. However*,* involvement largely depends on your understanding of the system. If you don’t understand the language and the system*,* you may not be included. Sometimes professionals do not engage you voluntarily*,* so it falls on the parents to demand involvement. This can be challenging for those who lack language skills or education to express their needs. I am happy with the help I receive and the way I am involved in my child’s care. This is possible because I am educated*,* understand the system and the language*,* and know my child’s needs.* (*Participant* 14)

### Theme 4. Late diagnosis (accessibility)

#### Neglect of early parental concerns

The experiences shared by immigrant parents bring attention to significant challenges in accessing timely diagnoses for their children with autism. Participants shed light on instances where healthcare and educational staff did not fully address parental concerns, leading to delays in evaluations and interventions. They expressed the difficulty of navigating a complex system where the progression through nursery evaluations, municipal decisions, and professional pedagogical team (PPT) assessments extends the timeline for diagnosis. These reflections suggest systemic processes that could be made more efficient to enhance early intervention efforts, which are vital for optimal developmental outcomes in children with autism. Moreover, one participant noted instances where health professionals did not fully recognize parental insights and observations regarding their child’s unique behaviors. The description of a three-year journey to obtain a diagnosis highlights areas where current systems might unintentionally delay access to support services. These parental narratives underscored the importance of considering structural adjustments within healthcare and educational systems to better incorporate the perspectives of immigrant parents, streamline referral processes, and minimize barriers to autism diagnosis. Such improvements have the potential to reduce parental stress and ensure that children receive timely and appropriate care and interventions crucial for their development.

*When parents raise concerns about their child*,* the nursery and health station often ignore them. I noticed my child was different when he was 2 years old and requested a referral to a specialist for a checkup*,* but they refused and said the nursery would follow up. Parents know their child best and want early intervention to receive the help they need. The nursery’s evaluation takes months*,* the municipality’s decision takes months*,* and the PPT evaluation process also takes months; altogether*,* it may take up to two years going from one place to another. Finally*,* they might refer you to the hospital*,* where it takes months to get an appointment and additional months to receive the diagnosis results. During this lengthy process*,* the child has no rights to receive any help*,* and for some parents*,* the entire process takes up to three years (Participant 14)*.

One parent shared her personal experience, explaining how her general practitioner (GP) initially ignored her concerns about her child’s development. Despite her repeated observations and requests for a referral to a specialist, the GP dismissed her worries and assured her that her child was fine. However, as time went on, the parent’s instincts proved to be accurate. After a long and difficult period of seeking help, her child was eventually diagnosed with autism. The early intervention services that could have greatly benefited her child were delayed due to the initial lack of response from her GP. Following the diagnosis, the doctor acknowledged his mistake and apologized to the parent for not addressing her concerns sooner.

*If my child had been diagnosed early and started therapy sooner*,* he would be much better now. Initially*,* my doctor did not listen to my concerns. Later*,* when my child was finally diagnosed*,* the doctor apologized for not addressing my concerns and diagnosing my child earlier. (Participant 4)*

#### Parents’ proposed improvements

Participants underscored the critical need for early autism interventions against the backdrop of increasing prevalence within the community. The participant’s account reveals a complex, multi-layered decision-making process that often results in prolonged waiting periods, some of which are deemed unnecessary. This highlights systemic inefficiencies that may impede timely intervention. The participant advocates for a more direct approach, where parents’ concerns are addressed by the experts who have the authority to make crucial decisions. Such direct engagement can facilitate a more thorough assessment of the child’s needs, expediting diagnosis and referrals for intervention. This stronger, streamlined process not only acknowledges the urgency of these developmental challenges but also respects the valuable insights parents bring to understanding their child’s unique needs. By refining this process, the healthcare system could significantly enhance outcomes, ensuring children receive the swift and appropriate support necessary for their development.

*The prevalence of autism is increasing in the community*,* and it is crucial for children to receive early intervention. However*,* the decision-making process has many layers*,* which can take a long time. Some of these waiting periods are unnecessary. Parents’ concerns should be addressed directly by the experts who make the final decisions. This way*,* these experts can evaluate the parents’ concerns*,* provide a diagnosis*,* and promptly refer the child for intervention (Participant 14)*.

## Discussion

This is the first study to explore immigrant parents’ experiences with autism intervention in Norway. Early intervention can improve children’s overall development, help them develop coping skills and strategies, and have long-term benefits that extend into adulthood [34, 35]. However, family risk factors such as socio-economic disadvantage and immigrant background are associated with a decreased likelihood of parents actively participating in intervention programs for their children [36].

Our study revealed that Somali and Eritrean parents in Norway were largely satisfied with the interventions their children received in terms of availability, accessibility, accommodability and acceptability. Satisfaction, as defined by Lebow, is the extent to which treatment meets the wants, wishes, and desires of clients [37]. Parent satisfaction was found to motivate parents to be actively involved in their child’s care [38]. Our findings align with a study in Poland, which reported above-average levels of satisfaction among parents [39]. The reported satisfaction among immigrant families may be influenced by the quality of services delivered. However, it is worth noting that East African parents come from countries where autism intervention barely exists. Thus, their ‘frame of reference’ to the conditions in their home countries may positively influence their perception of the services provided in Norway. Immigrants often compare their current situation to that in their home country [40].

Another factor potentially influencing the satisfaction levels among parents could be that nearly half of the study participants possessed university degrees, with the majority working in the health sector. These social connections and a high level of health literacy may help them navigate the complex healthcare system more effectively and feel satisfied with the services their children receive. A prior systematic review found that among immigrants, language skills and educational background are the most influential factors affecting health literacy [41]. However, one parent expressed dissatisfaction with the intervention due to limited contact and communication between himself and the school staff attending to his daughter. Research indicates that the least satisfied parents are those with minimal or no interaction with the professionals providing care to their child [42]. Overall, while the majority of parents were satisfied with the interventions, it’s essential for these programs to be tailored to individual needs and for clear communication to be established between parents and professionals, ensuring that realistic expectations are set and met.

Our study participants acknowledged that their children’s situation improved significantly because of the intervention provided to them. They expressed how essential the different interventions were for the development and growth of their children’s social skills, in particular, children’s communication and adaptive and social interactions. Research has shown that early interventions can have significant effects on children’s ability to function well [43]. In line with our study, parents who participated in earlier research expressed that early interventions were most effective for their children when provided at an early age, and parents also believed that the intervention was most beneficial when there was a consistent and collaborative nature to the intervention among home, school, and all team members [44]. In line with prior research [45], most parents in our study indicated that the age at which the intervention began is important, and they felt that the earlier the intervention began, the better the results.

The study participants reported that the intervention met parents’ expectations, as evidenced by its positive effect on the child’s outcomes, underscoring the acceptability of services. This alignment with parental expectations highlights that when interventions are designed to meet anticipated standards and visibly improve child development, it increases the overall acceptability of the healthcare services provided, fostering greater satisfaction and trust among families [46]. Parents’ expectations of what they hope to achieve through intervention ranged widely from improvement in speech, communication and social interactions to achieving normal. A prior study stated that ‘parental optimism and expectations for improvement through intervention may directly influence parental health and well-being, with potential carryover effects to improved child outcomes’ [26]. As parents are primary caretakers to mental healthcare for their children, their expectations that the intervention is ineffective may undermine help-seeking, retention, and response [47]. Although most of the parents in our study reported that the intervention met their expectations, one of the parents doubted the quality of the services. Prior study reported that immigrant families are at greater risks to receive poorer quality services, including longer waiting times and interventions that lack culturally sensitive or research-based best practices [48]. There is a consensus that parents’ satisfaction levels are optimal when they had easy access to the intervention services, receive sufficient information and support, proper training in applying therapy exercises at home, and perceived respect and support from service providers [49].

The parents in our study reported that collaboration between the staff providing the intervention and the parents was good, reflecting the importance of accommodability in services that effectively integrate family involvement. This positive collaboration suggests that when services are accommodable, valuing parental insights and fostering cooperative relationships, they can significantly enhance the intervention process and satisfaction, underscoring the need for healthcare systems to maintain flexibility and responsiveness to individual family dynamics. The active participation of parents in interventions ensures that the child’s specific needs are met comprehensively [50]. A study revealed a positive relationship between parental involvement in the intervention and improved social behaviour in children with ASD, which suggests that children with ASD demonstrate more positive behaviours if parents are more involved in the intervention [50]. Our study found that parents received practical training on how to bring up a child with autism. This training not only educates parents on how to treat children with ASD, but also teaches them important skills for navigating the challenges that may arise. By being trained about ASD, parents demonstrated that they are better equipped to make informed decisions, advocate for their child’s needs, and collaborate effectively with professionals to provide the best care for their child.

Despite parents’ satisfaction with the interventions provided for their children, they faced significant challenges in accessing prompt autism diagnoses, highlighting key accessibility issues within the healthcare system. This delay in diagnosis hindered timely intervention and support opportunities for their children. When parents observed their child’s abnormal development, they reported these concerns to healthcare providers. Unfortunately, their concerns were often dismissed rather than being acknowledged and addressed. According to previous literature, there are two main factors contributing to delays in autism diagnosis [51]. First, clinicians may mistakenly attribute autism symptoms to other health issues. Second, cultural differences may affect how likely parents are to notice and report early developmental concerns to clinicians [48]. Our findings align with numerous studies that highlight parents’ dissatisfaction with the diagnostic process, specifically due to the extended timeframe between initial concerns and the formal diagnosis [51–53]. Additionally, large survey studies have shown that parents experience an average delay of 3.5 years between their initial expressions of concern and receiving a formal diagnosis, which they perceive as excessively prolonged [54]. Consistent with our findings, a study conducted in the Netherlands revealed that first-line healthcare workers often perceive parental concerns about autism spectrum disorder (ASD) as less severe than the parents themselves do, contributing to diagnostic delays [53]. For parents, discovering their child’s significant developmental issues is a distressing experience, which becomes even more challenging when healthcare providers dismiss their concerns [55]. Participants in our study reported feeling that their concerns were often ignored, dismissed, or judged as exaggerated. An earlier study similarly found that parents felt worn out discussing their initial concerns due to a fear of being ignored or because professionals advised waiting rather than validating their observations [56]. The benefits of early diagnosis of autism include the opportunity for early entry of children into interventions, which have been shown to enhance developmental outcomes and improve adaptive skills, while it enables parents to access appropriate support services and educational resources [57]. Given parents’ concerns about the delay in diagnosis and the importance of prompt entry of children in appropriate and comprehensive interventions, it is important for health providers and healthcare systems in Norway to establish a parent friendly approach for the diagnosis of ASD among immigrant children.

This study has both strengths and limitations. The 15 in-depth interviews with parents from Eritrea and Somalia, including both mothers and fathers, added substantial depth and richness to the results. The strength of the study was underscored by its rigorous validation processes, which included verifying the authenticity of participant quotes through a meticulous review of interviews. This ensured that the participants’ words were accurately captured [33]. Furthermore, the coding process was subjected to thorough examination, with two researchers independently cross-checking and mutually agreeing upon the codes, thereby enhancing the reliability of the analysis. Peer reviews and iterative feedback mechanisms played crucial roles in refining the study, contributing to the robustness, supportability, and credibility of the conclusions drawn [33]. A notable weakness of the study was that, although the ages of children with autism ranged from 4 to 24 years, it did not investigate how parental experiences with healthcare might vary according to the age of their children. This omission may have concealed important insights into how healthcare needs and challenges differ across developmental stages. Additionally, most study participants had university education, which means the results may not be fully applicable to parents with little or no education or those who do not speak Norwegian fluently. Given that education and language competence can significantly influence awareness, access to information, and the ability to navigate healthcare systems [41], the experiences and challenges articulated by the well-educated participants may differ markedly from those faced by less educated parents. As a result, the study findings may not fully capture the diversity of experiences within the broader Somali and Eritrean communities in Norway, potentially limiting the applicability of the insights to all demographic segments within these populations. Future research should aim to include a more varied participant pool, encompassing a broader spectrum of educational backgrounds, to ensure a more comprehensive understanding of the issues at hand. Furthermore, investigating healthcare providers’ perspectives on diagnosing children with autism is of paramount importance.

## Conclusion

The study participants reported enduring lengthy waiting periods for autism diagnoses, which can significantly heighten stress and anxiety levels. Such delays are particularly detrimental as they postpone the child’s access to crucial interventions. It is vital for healthcare providers to promptly and effectively respond to the developmental concerns expressed by immigrant parents. By enhancing services to be more parent-friendly, the overall healthcare experience can be improved, especially regarding the timely diagnosis of autism.

## Supplementary Information

Supplementary Material 1.

Supplementary Material 2.

## Data Availability

Data is provided within the manuscript.
