# Supplementary material for: Somali and Eritrean parents experiences and challenges in the diagnosis and early intervention of autism spectrum disorder in Norway
Source: BMC Pediatr. 2026 Mar 26;26:408. doi: 10.1186/s12887-026-06765-y (PMC13141427; doi:10.1186/s12887-026-06765-y)
Supplement: Supplementary file 1 — Supplementary Material 1. [file 12887_2026_6765_MOESM1_ESM.pdf]

Question guide

Relation' mother or father

Education?

Country of birth?

How many children born in Norway and how many were born in your home country?

How many, of your children, have been diagnosed with autism?

Did the child\children with autism born in Norway or your home country?

Age of the child\children with autism?

According to you, what would be the cause of your child's difficulties?

How did you find out that your child might be having difficulties?

What kind of treatment or intervention do you think your child should receive?

How are your child's difficulties perceived in your native culture?

Can you tell me about Early Intensive Behavioral Intervention (EIBI) provided to your child? How do you see the intervention? How has the intervention affected the situation of your child?

Did the intervention meet your needs and expectations? What do you think has been done in accordance with your needs and expectations? What do you think has not been done as per your needs and expectations?

How was your involvement in provision of EIBI to your child?

Did your viewpoints taken into consideration during the intervention? If not, what do you think was the reason?

What do you think would have been done better if your viewpoints were considered?

How is your satisfaction with the intervention in general as regards to the collaboration between you and the service providers?

In general, how can you describe the importance of the EIBI to your child? What do you think, (if any) are the barriers to tailoring the intervention to your needs and expectations?

What do you think should be done (recommendation) to acclimatize the intervention to your needs and expectations?
